# Knowledge, Attitudes, and Practices regarding Diarrhea and Cholera following an Oral Cholera Vaccination Campaign in the Solomon Islands

**DOI:** 10.1371/journal.pntd.0004937

**Published:** 2016-08-22

**Authors:** Eleanor Burnett, Tenneth Dalipanda, Divi Ogaoga, Jenny Gaiofa, Gregory Jilini, Alison Halpin, Vance Dietz, Kashmira Date, Eric Mintz, Terri Hyde, Kathleen Wannemuehler, Catherine Yen

**Affiliations:** 1 Centers for Disease Control and Prevention, Atlanta, Georgia, United States of America; 2 Ministry of Health and Medical Services, Honiara, Solomon Islands; 3 Gizo Hospital, Gizo, Solomon Islands; Massachusetts General Hospital, UNITED STATES

## Abstract

**Background:**

In response to a 2011 cholera outbreak in Papua New Guinea, the Government of the Solomon Islands initiated a cholera prevention program which included cholera disease prevention and treatment messaging, community meetings, and a pre-emptive cholera vaccination campaign targeting 11,000 children aged 1–15 years in selected communities in Choiseul and Western Provinces.

**Methodology and Principal Findings:**

We conducted a post-vaccination campaign, household-level survey about knowledge, attitudes, and practices regarding diarrhea and cholera in areas targeted and not targeted for cholera vaccination. Respondents in vaccinated areas were more likely to have received cholera education in the previous 6 months (33% v. 9%; p = 0.04), to know signs and symptoms (64% vs. 22%; p = 0.02) and treatment (96% vs. 50%; p = 0.02) of cholera, and to be aware of cholera vaccine (48% vs. 14%; p = 0.02). There were no differences in water, sanitation, and hygiene practices.

**Conclusions:**

This pre-emptive OCV campaign in a cholera-naïve community provided a unique opportunity to assess household-level knowledge, attitudes, and practices regarding diarrhea, cholera, and water, sanitation, and hygiene (WASH). Our findings suggest that education provided during the vaccination campaign may have reinforced earlier mass messaging about cholera and diarrheal disease in vaccinated communities.

## Author Summary

We assessed knowledge, attitudes and practices of diarrhea and cholera disease and prevention in two areas of the Solomon Islands near Papua New Guinea. Both areas were ‘at risk’ for cholera disease and received messages about cholera prevention. Later, one of the areas also received vaccination against cholera. This was the first time cholera vaccine was administered to a population that had never reported cholera. Our survey found that people living in the area were cholera vaccine was administered were more likely to know the signs and symptoms and treatment of cholera, as well as be aware of cholera vaccine. We think this could be related to the extra education provided with vaccination. This was the first knowledge, attitudes, and practices survey about diarrhea and cholera disease and prevention and prevention in a population that had not been exposed to cholera.

## Introduction

**Fig 1:**
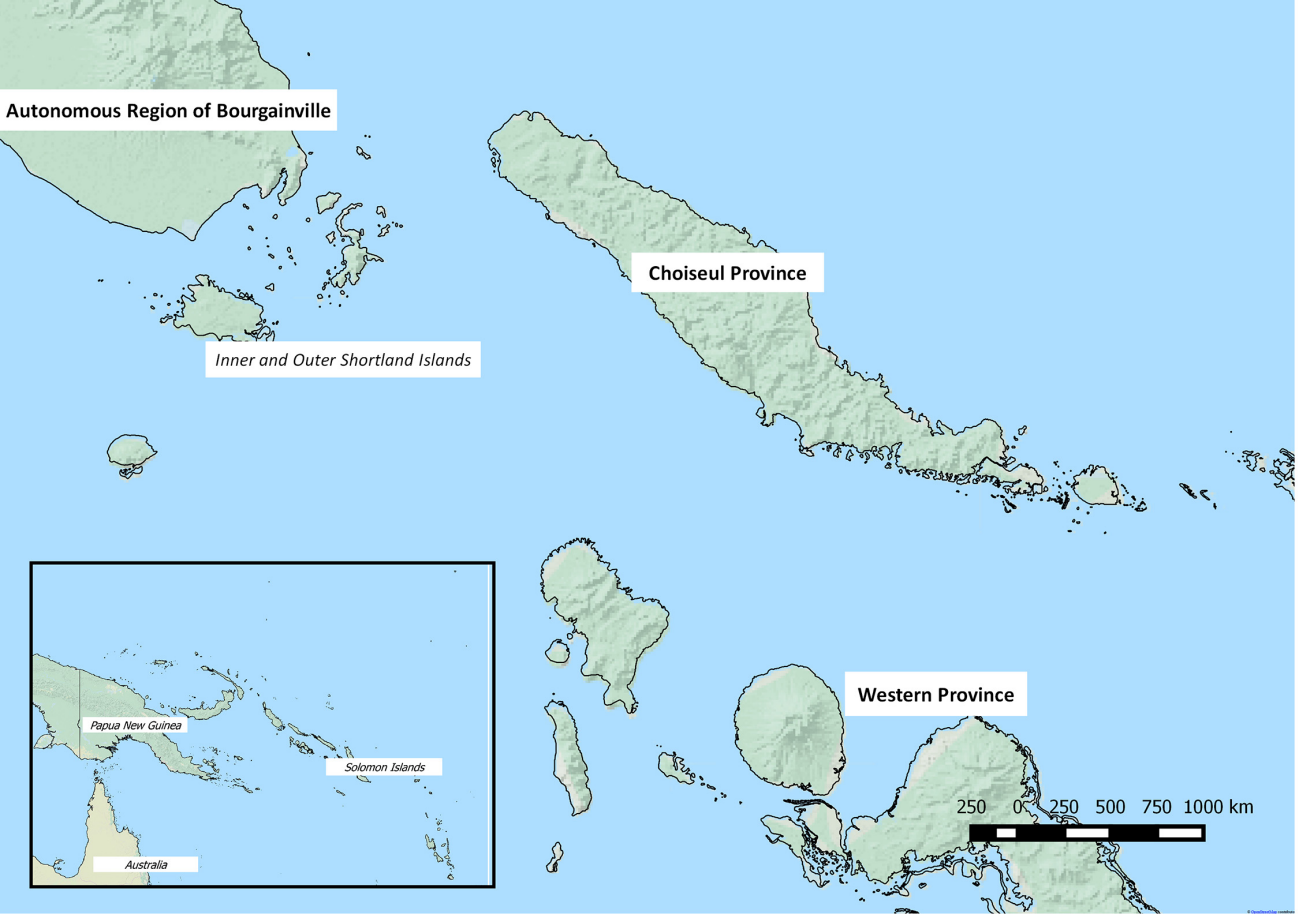
Map of Papua New Guinea and the Solomon Islands [2].

From July 2009 until late 2011, an outbreak of cholera in Papua New Guinea (PNG) resulted in >15,500 cases and >500 deaths in 8 of 20 province-level divisions and the Autonomous Region of Bougainville, situated in the western archipelago of the Solomon Islands in the South Pacific (Fig 1) [1]. At the time of the outbreak, no cholera was confirmed in the Solomon Islands, a country of approximately 560,000 people and nearly 1,000 islands [3]. However the risk for cholera introduction and transmission was considered high, due to geographical location, frequent travel between Bougainville and the Solomon Islands, and limited access to improved sources of water and improved sanitation infrastructure in the Solomon Islands. As a result, the Government of the Solomon Islands initiated a cholera prevention program in the two provinces adjacent to Bougainville: Choiseul and Western Provinces.

In April 2011, the Ministry of Health and Medical Services (MHMS) began a messaging campaign in both Provinces about cholera disease, transmission, and prevention via radio programs, newspapers, and community meetings (personal communication, MHMS). Subsequently, the MHMS, with support from the World Health Organization (WHO), the United Nations Children’s Fund (UNICEF), and the Australian Agency for International Development (AusAID), conducted a cholera vaccination campaign for 11,000 children 1 to 15 years of age in selected communities in Choiseul Province and the Inner and Outer Shortland Islands of Western Province; children were brought to vaccination sites by their parents, and age was verified by date of birth. For the campaign, the MHMS procured Shanchol (Shantha Biotechnics Ltd., India), an oral, whole-cell, killed vaccine administered in two doses at least two weeks apart [4]. Vaccination teams stationed at health centers and nurse aid posts administered 11,888 doses in May 2012 and 11,318 doses in July 2012 [5] and provided messages about safe water, sanitation, and hygiene practices verbally and through brochures and posters.

This cholera vaccination campaign was the first use of Shanchol in a pre-emptive campaign in an area that had never reported cholera and provided a unique opportunity to assess the impact of messaging on cholera-naive communities. To test our hypothesis that education during the cholera vaccination campaign may have reinforced the earlier, wider messaging campaign, we conducted knowledge, attitude, and practices (KAP) survey related to diarrhea and cholera in vaccinated and unvaccinated communities.

## Methods

### Sampling and study population

During 6–10 December 2012, approximately 5 months after the cholera vaccination campaign had ended, we conducted a household-level cross-sectional survey in communities targeted for vaccination (Choiseul Province and the Shortland Islands of Western Province), subsequently referred to as “vaccinated areas,” and in communities on nearby islands of Western Province not targeted for cholera vaccination, referred to as “unvaccinated areas.” These populations were chosen because they were both considered at-risk for cholera importation and were both targeted during the initial information campaign. We randomly sampled six wards each in vaccinated and unvaccinated areas, and then randomly sampled two villages within each ward. Due to small village sizes, we interviewed a convenience sample of 12 households in each selected village. Households that participated in a previous cholera KAP survey were excluded. In areas where there was civil unrest, the survey team approached the village closest to the one originally selected.

### Data collection

Before the survey, team members were trained on data collection methods and survey questions were translated into Pidgin, the main language of the Solomon Islands. For each household, survey teams interviewed the female head of household or an alternate adult ≥18 years old and collected information regarding household demographic and socioeconomic characteristics; recent diarrheal illness; knowledge, attitudes, and practices (KAP) related to diarrhea and cholera; water source, storage, and handling practices; routine and cholera vaccines; hand hygiene and sanitation practices; and vaccine accessibility. Survey teams also made observations regarding water storage and the presence or absence of areas for handwashing, soap, and latrines/toilets.

### Statistical analyses

Data were entered into an Access database and analyzed using SAS v9.3. Frequencies and percentages were calculated for categorical variables; median and IQR were calculated for continuous variables. Univariate logistic regression models were fit to evaluate whether there were significant differences in KAPs between vaccinated and unvaccinated areas using generalized estimating equations to account for the ward-level clustering; generalized score statistic p-values ≤0.05 were considered significant.

This evaluation was reviewed by the National Health Research and Ethics Committee of Solomon Islands Ministry of Health and Medical services and Centers for Disease Control and Prevention and considered non-research.

## Results

We interviewed 108 households in vaccinated areas and 173 households in unvaccinated areas. Respondents from 46 households reported that they had participated in an earlier cholera KAP survey. Since we could not determine whether specific respondents had participated in the previous survey and had been exposed to similar questions, we excluded them from the analysis. Thus, the final analysis included 89 households in vaccinated and 146 households in unvaccinated areas (Table 1). In both areas, the respondents’ median age was 39 years, and median household size was six individuals. The reported literacy rate of respondents was high in both vaccinated (91%) and unvaccinated (98%) areas. A higher proportion of households in vaccinated areas (17%) than in unvaccinated areas (5%) reported at least one household member of any age having had diarrhea in the previous week (p = 0.01).

**Table 1:** Self-reported characteristics of individual and household survey respondents in communities targeted and not targeted for vaccination. Solomon Islands, 2012.

	No. (%) in areas targeted for vaccination	No. (%) in areas not targeted for vaccination	p-value
	(n = 89)	(n = 146)	
**Individual-level**			
Age of respondent, median(IQR)	39 (31–44)	39 (30–49)	
Female respondent	64 (72%)	74 (51%)	0.6
Able to read and write	81 (91%)	142 (98%)	0.1
Primary education or less	51 (57%)	59 (40%)	0.08
**Household-level**			
People/hh, median (IQR)	6 (4–8)	6 (4–8)	
Diarrhea in the last week	15 (17%)	7 (5%)	*0*.*009*
Electricity	46 (59%)	112 (78%)	0.06
Cooking gas	1 (1%)	8 (6%)	0.06
Radio	25 (32%)	48 (34%)	0.4
Cell phone	29 (37%)	97 (68%)	*0*.*01*
Boat	20 (25%)	18 (13%)	0.06
Fishnet	10 (13%)	19 (13%)	1.0

Nearly all respondents correctly named at least one cause (97% in vaccinated areas, 98% in unvaccinated areas) and one treatment (97% in vaccinated areas, 98% in unvaccinated areas) of diarrhea (Table 2). The most commonly mentioned cause was ‘poor hygiene,’ and treatment was ‘go to clinic’. When asked to name a diarrhea prevention measure, 88% of respondents in vaccinated areas provided a correct answer compared with 99% in unvaccinated areas (p = 0.04). In both, the most commonly mentioned prevention strategies were ‘hand washing’, ‘clean cooking utensils’, and ‘cover food to keep away from flies’. Respondents in vaccinated areas were more likely to report recent education about diarrhea (37% in vaccinated, 14% in unvaccinated; p = 0.02). In both areas, the most common source of diarrhea information was a community health worker or clinician.

**Table 2:** Knowledge of causes, prevention, and treatment of diarrhea and cholera in areas targeted and not targeted for OCV vaccination. Solomon Islands, 2012.

	**Targeted**		**Not targeted**		p-value
	(n = 89)		(n = 146)		
	n	%	n	%	
**Diarrhea**					* *
≥1 correct cause named^1^	86	97%	143	98%	0.5
≥1 correct prevention measure named^2^	78	88%	144	99%	*0*.*04*
≥1 correct treatment named^3^	86	97%	143	98%	0.6
Received education about diarrhea prevention or treatment within the past 6 months	33	37%	20	14%	*0*.*02*
Aware of cholera	67	75%	64	44%	0.09
	(n = 67)		(n = 64)		p-value
**Cholera**	n	%	n	%	
≥1 correct cause named^4^	37	55%	17	27%	0.1
≥1 correct symptom named^5^	43	64%	14	22%	*0*.*02*
≥1 correct prevention measure named^6^	45	67%	22	34%	0.1
≥1 correct treatment named^7^	64	96%	32	50%	*0*.*02*
Received education about cholera prevention or treatment within the past 6 months	22	33%	6	9%	*0*.*04*

^1^ Drinking bad water, eating bad food, unwashed fruits/vegetables, flies/insects, poor hygiene

^2^ Wash hands with soap and water, cook food thoroughly, boil water, wash fruits/vegetables, clean cooking utensils/vessels, treat water, drink cooled, boiled water, dispose of human waste properly, cover food to keep away from flies

^3^ Go to clinic/hospital, use oral rehydration solution/salt-sugar solution, go to a traditional healer, coconut-salt solution

^4^ Drinking bad water, eating bad food, unwashed fruits/vegetables, flies/insects, poor hygiene

^5^ Fever, vomiting, watery diarrhea, stomach/abdominal pain, bloody diarrhea, dehydration

^6^ Wash hands with soap and water, cook food thoroughly, boil water, wash fruits/vegetables, clean cooking utensils/vessels, treat water, drink cooled, boiled water, dispose of human waste properly, cover food to keep away from flies

^7^ Go to clinic/hospital, use oral rehydration solution/salt-sugar solution, go to a traditional healer, coconut-salt solution

In vaccinated areas, 75% of respondents were aware of cholera compared with 44% in unvaccinated areas (p = 0.09). Among respondents aware of cholera, 55% of respondents in vaccinated areas correctly named at least one cause of cholera transmission, compared with 27% of respondents in unvaccinated areas (p = 0.10); similarly, 67% in vaccinated and 34% in unvaccinated areas named one prevention measure (p = 0.15). Those in vaccinated areas were more likely than those in unvaccinated areas to correctly name at least one sign or symptom (64% vs. 22%; p = 0.01), identify watery diarrhea as a sign (57% v. 17%; p = 0.01), and name at least one treatment (96% vs. 50%; p = 0.02) for cholera. Persons in vaccinated areas were also more likely than those in unvaccinated areas to report any recent education about cholera (33% vs. 9%; p = 0.04). Among cholera-aware respondents in vaccinated areas, the most commonly mentioned sign of cholera was ‘watery stool’ (57%); the most commonly mentioned cause was ‘poor hygiene’ (39%); the most commonly mentioned prevention was ‘hand washing’ (42%); and the most commonly mentioned treatment was ‘go to clinic’ (90%).

In a subanalysis of unvaccinated households, 40 of 58 (69%) unvaccinated households in areas targeted for vaccination reported awareness of cholera while 64 of 144 (44%) unvaccinated households in areas not targeted for vaccination reported awareness. Though the difference was not statistically significant (p = 0.21), these results suggests that cholera messaging may have reached more households in areas targeted for the OCV campaign, even if they did not include any vaccine recipients. Unvaccinated but cholera-aware households in targeted areas were more likely to report watery diarrhea as sign of cholera than unvaccinated but cholera-aware households in areas not targeted (48% v. 17%; p = 0.01), to know any signs or symptoms of cholera (58% v. 21%; p = 0.03), and to know any treatment for cholera (98% v. 50%; p = 0.01). There were no differences in knowledge of causes (p = 0.33), knowledge of prevention (p = 0.27), or recent cholera education (p = 0.50) between the two groups.

Drinking water sources were similar in vaccinated and unvaccinated areas, with only 17% and 10% respectively reporting an unprotected surface source or well as their main source of drinking water. However, a greater proportion of those surveyed in vaccinated areas reported ever treating their drinking water (53% vaccinated, 11% unvaccinated; p = 0.03) (Table 3). In both groups, fewer than 50% (47% vaccinated, 39% unvaccinated) reported regularly washing their hands. Eighty-five percent of households in vaccinated and 61% in unvaccinated areas reported their usual toilet facilities as the ocean or bush (p = 0.05).

**Table 3:** Reported and observed water source, storage, and handling practices of an adult household member in areas targeted and not targeted for oral cholera vaccination. Solomon Islands, 2012.

	Targeted		Not targeted		p-value
	(n = 89)		(n = 146)		
	n	%	n	%	
Unprotected drinking water source^1^	15	17%	14	10%	0.7
Unprotected non-drinking water source^1^	45	51%	29	20%	*0*.*04*
Primary water source ever unavailable	35	39%	80	55%	0.3
Ever treats drinking water	47	53%	17	12%	0.3
Regularly washes hands	42	47%	58	39%	0.4
Soap in household^2^	88	99%	143	98%	0.6
Soap at handwashing area^3^	21	24%	60	41%	0.1
Ocean or bush toilet facilities^4^	76	85%	89	61%	*0*.*05*

^1^ Unprotected well, river, stream, or lake

^2^ Reported

^3^ Observed

^4^ Reported household members use ocean or bush as usual toilet facilities

Acceptance of routine childhood vaccines was high in both vaccinated and unvaccinated areas: in only one household were all members completely unvaccinated; however, about a quarter of respondents reported having a concern about vaccination for themselves or their child. The most common concerns were related to side effects. In vaccinated areas, more respondents had heard of cholera vaccine compared with unvaccinated areas (48% vs. 14%; p = 0.02). Nearly all participants reported they would get the cholera vaccine for themselves, if available (97% in vaccinated, 98% in unvaccinated). In vaccinated areas, 35% of respondents reported that at least one household member 1–15 years of age had received the cholera vaccine, though only 21% had an OCV campaign card. In unvaccinated areas, 2 (1%) households reported that at least one member had received OCV.

## Discussion

To the best of our knowledge, this is the first household-level evaluation to assess knowledge, attitudes, and practices regarding diarrhea, cholera, and water, sanitation, and hygiene (WASH) in the setting of a pre-emptive OCV campaign in a cholera-naïve community. We found that knowledge of causes and treatments for diarrhea were high both in areas targeted and not targeted for cholera vaccination, while knowledge of cholera vaccine and cholera signs and symptoms and treatments was higher in areas that were targeted for vaccination despite similar levels of cholera awareness in both areas. This was true among only households that were not vaccinated as well as all households that participated. The reason for these findings may be that both vaccinated and unvaccinated areas had previously received messages about cholera disease, prevention and treatment, but key messages were likely reiterated during the vaccination campaign. Survey respondents also reported high acceptance of OCV. This finding is supported by the successful implementation of the vaccination campaign by MHMS, with administrative OCV coverage of 108% and 102% during the first and second rounds of the campaign, respectively (personal communication, MHMS); there was no additional assessment of vaccination coverage associated with this campaign, and the high coverage estimates may be due to population movement unaccounted for in official population estimates. Similar knowledge of cholera and attitudes toward OCV have also been reported in Dhaka, Bangladesh, an endemic setting [5]. Of note, use of water treatment practices, hand washing, and use of improved toilet facilities were low both in targeted and non-targeted areas, even though knowledge of diarrhea prevention and treatment was high among survey respondents and most households had protected sources of drinking water. Reasons for this are unknown and should be investigated further.

Another interesting finding was that knowledge of diarrhea was higher than knowledge of cholera among cholera-aware respondents in vaccinated areas; 97% and 55%, respectively, correctly listed a cause of diarrhea and cholera. Although documentation of health messages provided during the prevention and vaccination campaign is limited, this finding would be expected if cholera prevention messages had been framed as ‘diarrhea’ prevention. A recent KAP survey in Thailand reported that building on diarrhea knowledge while distinguishing cholera was a key challenge in effective communication (personal communication, H. Scobie). Our finding also suggests an opportunity to improve future cholera awareness campaigns by capitalizing on previous familiarity with diarrheal disease prevention and treatment.

This survey had several limitations. Most importantly, the unvaccinated group may not have been comparable to the vaccinated group, as evidenced by the higher proportion of households in vaccinated areas reporting diarrhea in the previous week; this may represent differences in socio-economic status as well as more familiarity with diarrhea and long-term exposure to WASH messages. Interviewers were aware which areas were targeted for vaccination, which may have introduced additional bias during data collection. Additionally, the small size and convenience sampling in a cross-sectional survey limits the generalizability of these findings and limits the precision as well as the power to detect differences among the population groups. Also, the exclusion of 46 households that had participated in an earlier KAP survey may have biased results. However, a sub-analysis comparing excluded households with included households found no major differences in socioeconomic status or in cholera awareness with other households in the same province. Finally, the limited documentation of specific health messages provided before and during the vaccination campaign prevented more specific comparisons about diarrhea and cholera knowledge. We did find that awareness and specific knowledge was high, however we are unable to determine which specific messages and modes of communication were most effective. Improved documentation of the messaging and other cholera prevention interventions would have allowed us to assess messages for effectiveness and potentially to identify gaps that may have explained our findings.

As of this writing, no suspected cholera cases have been reported from any part of Solomon Islands. Therefore, behavior change and improved infrastructure should remain a priority to effectively prevent cholera and other diarrheal disease. Future cholera prevention campaigns in previously unexposed communities should hone in on effective messages that capitalize on diarrheal knowledge. Additionally, learning from the limitations of this survey, future evaluations of cholera prevention messaging and vaccination campaigns could be improved by having clearly documented messages, modes of communications, and timeframes as well as a complete assessment of coverage in the target population when feasible.

## References

[1] Horwood PF, Mueller KS, Jonduo MH et al Spatio-temporal epidemiology of the cholera outbreak in Papua New Guinea, 2009–2011. BMC Infect Dis. 2014; 14:449 doi: 10.1186/1471-2334-14-449 25141942 10.1186/1471-2334-14-449 PMC4158135

[2] QGIS Development Team. QGIS Geographic Information System. Open Source Geospatial Foundation Project 2015 http://qgis.osgeo.org.

[3] World Bank. World development indicators, Solomon Islands. The World DataBank http://databank.worldbank.org/data/views/reports/tableview.aspx (accessed on 14 5 2014).

[4] World Health Organization. Cholera vaccines: WHO position paper. Wkly Epidemiol Rec. 2010; 85:117–28. 20349546

[5] Wahed Tasnuva, et al "Knowledge of, attitudes toward, and preventive practices relating to cholera and oral cholera vaccine among urban high-risk groups: findings of a cross-sectional study in Dhaka, Bangladesh." BMC public health 13 1 (2013): 242. 23509860 10.1186/1471-2458-13-242 PMC3608226

